# Modeling Alzheimer’s disease with iPSC-derived brain cells

**DOI:** 10.1038/s41380-019-0468-3

**Published:** 2019-08-07

**Authors:** Jay Penney, William T. Ralvenius, Li-Huei Tsai

**Affiliations:** Department of Brain and Cognitive Sciences, Picower Institute for Learning and Memory, Massachusetts Institute of Technology, Cambridge, MA 02139 USA

**Keywords:** Neuroscience, Molecular biology, Cell biology, Diseases, Genetics

## Abstract

Alzheimer’s disease is a devastating neurodegenerative disorder with no cure. Countless promising therapeutics have shown efficacy in rodent Alzheimer’s disease models yet failed to benefit human patients. While hope remains that earlier intervention with existing therapeutics will improve outcomes, it is becoming increasingly clear that new approaches to understand and combat the pathophysiology of Alzheimer’s disease are needed. Human induced pluripotent stem cell (iPSC) technologies have changed the face of preclinical research and iPSC-derived cell types are being utilized to study an array of human conditions, including neurodegenerative disease. All major brain cell types can now be differentiated from iPSCs, while increasingly complex co-culture systems are being developed to facilitate neuroscience research. Many cellular functions perturbed in Alzheimer’s disease can be recapitulated using iPSC-derived cells in vitro, and co-culture platforms are beginning to yield insights into the complex interactions that occur between brain cell types during neurodegeneration. Further, iPSC-based systems and genome editing tools will be critical in understanding the roles of the numerous new genes and mutations found to modify Alzheimer’s disease risk in the past decade. While still in their relative infancy, these developing iPSC-based technologies hold considerable promise to push forward efforts to combat Alzheimer’s disease and other neurodegenerative disorders.

## Introduction

Alzheimer’s disease (AD) is a devastating and ultimately fatal form of neurodegeneration characterized by progressive loss of cognition and disruption of basic functions, such as swallowing, walking, attention, and memory [1]. As the sixth leading cause of death in the USA, the disease places a tremendous emotional and financial burden on families, caregivers, and the health care system [2]. Projections show that this burden will grow as domestic and world populations continue to age [3]. AD has been the subject of intense research efforts over the past 40 years, with human genetic studies identifying numerous mutations that either cause, or alter risk for the disease. In parallel, cellular and rodent models have been workhorses in deepening our understanding of numerous pathophysiological mechanisms associated with disease progression. Despite these efforts, there remains no cure for AD. The only current FDA approved treatments for AD target cholinergic and/or glutamatergic neuronal function, providing modest and transient cognitive benefit, but do not alter disease course or underlying neurodegeneration [4, 5]. The continued failure of promising therapeutics to provide benefit in human patients points to the need for improved model systems that better mimic the pathophysiology of AD patients. The advent in the past decade of techniques to generate human induced pluripotent stem cells (iPSCs) and to differentiate them into the various cell types of the body, including brain cells, has ushered in a new era of neurodegenerative disease research that offers renewed hope in tackling an old problem. Here we provide an overview of iPSC-based models of AD that have been developed, or are being developed, as well as highlighting important research directions going forward.

## Alzheimer’s disease

First described by Alois Alzheimer in 1907, AD is characterized by progressive cognitive decline, initially and particularly, affecting short-term memory, but later also language, mood, movement, and physiological functions [1]. Early onset familial AD (FAD) strikes in the fourth or fifth decade of life, while sporadic late onset AD (SAD) typically develops after the age of 70. The pathological hallmarks of AD—shared by FAD and SAD—are senile plaques and neurofibrillary tangles (NFTs), accompanied by progressive neurodegeneration [6]. Plaques are extracellular aggregates composed largely of amyloid-β (Aβ) peptides, while NFTs are intracellular inclusions rich in hyperphosphorylated tau protein. Various forms of Aβ induce cellular dysfunction and toxicity in vitro and in vivo. Likewise, mutated or hyperphosphorylated tau species are prone to aggregation and can cause neuronal dysfunction and cell death. Intriguingly, while both Aβ and tau can exhibit toxicity independently, their pathologies are linked; numerous studies show that Aβ buildup can induce tau hyperphosphorylation [7]. Other common pathological signs of AD include gliosis, inflammation, blood–brain barrier (BBB) disruptions, metabolic disturbances, altered endocytic and cellular degradation pathways, and elevated DNA damage [8]. The relative importance and interconnections between these phenomena remain subjects of intense research.

**Fig. 1:**
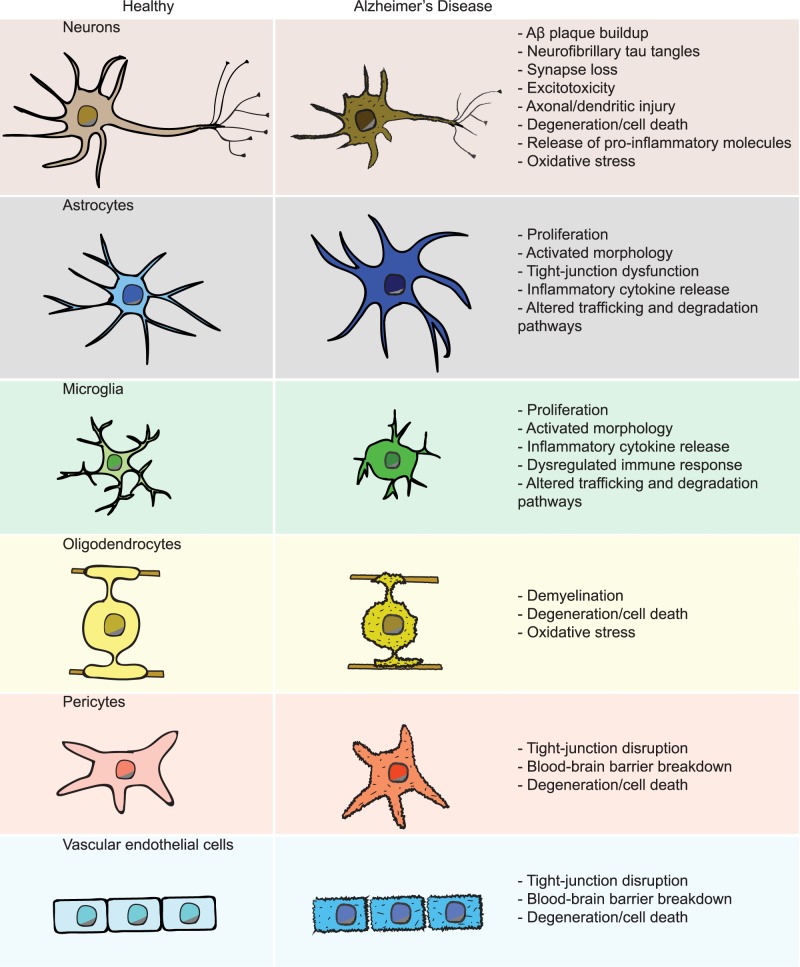
Brain cell types in Alzheimer’s disease. A summary of the major human brain cell types and the alterations they exhibit in AD

Following many years of focus on neuron-based mechanisms of neurodegeneration in AD, recent genetic studies have shifted attention to a more holistic view incorporating the functions of multiple different cell types of the brain [8, 9] (Fig. 1). Neuron dysfunction and degeneration undoubtedly underlies a large part of the characteristic cognitive decline during AD, but the brain also consists of many other non-neuronal cell types [8, 10, 11]. These cells are increasingly being recognized for helping to maintain proper brain function as well as ensuring the long-term health and survival of neurons. Oligodendrocytes insulate neuronal axons and promote fast synaptic transmission [12]. Astrocytes and microglia closely associate with synapses, the sites of communication between neurons, to form what is known as the “tripartite synapse” [13, 14]. Astrocytes, in addition to playing critical roles in neurotransmitter recycling, also perform important metabolic functions in the brain [15]. Microglia, the resident innate immune cells of the brain, are involved in the pruning of synapses, in particular during development but also in the context of adult synaptic plasticity [16]. Throughout life, microglia also act as sentinels that detect and remove exogenous and endogenous invaders and debris, including pathogens, dying brain cells, cancerous cells and protein aggregates such as Aβ plaques [17, 18]. All of the mentioned glial cell types can also contribute trophic support for other brain cells, secrete pro- or anti-inflammatory factors, and participate in clearance of toxic substances from the brain milieu [8]. Aside from glia, the cells of the BBB form a physical impediment to the passage of most cells and molecules between the central nervous system and peripheral blood circulation. These specialized blood vessels are composed of vascular endothelial cells (VECs), pericytes, and astrocytes that can break down during AD and exacerbate its progression [19]. The functions and contribution of each of these non-neuronal brain cell types to AD-associated neurodegeneration remains incompletely understood.

FAD exhibits an early onset of symptoms and is dominantly inherited, which facilitated the identification in the early 1990’s of disease-causing mutations in three different genes, encoding the amyloid precursor protein (APP), presenilin 1 (PSEN1), and presenilin 2 (PSEN2) [20–22]. It was already known that the Aβ peptide is a major constituent of amyloid plaques, and is derived from sequential proteolytic cleavage of APP [23]. Subsequent studies revealed that PSEN1 and PSEN2 are components of the gamma secretase complex, which carries out amyloidogenic cleavage of APP to produce Aβ peptides ranging from 36 to 43 amino acids in length. Aβ_40_ and Aβ_42_ are the most common isoforms, with longer forms such as Aβ_42_ and Aβ_43_ being more aggregation prone [24]. AD-linked mutations in APP, PSEN1, and PSEN2, as well as APP duplications, all increase either the total amount of Aβ_42_, or the ratio of Aβ_42_/Aβ_40_ produced by neurons [25, 26].

Together with the observation that amyloid buildup is a prominent event in disease pathology, these genetic findings laid the foundation for the “amyloid cascade hypothesis” of AD, placing neuron-derived Aβ at the top of a cascade ultimately leading to neurodegeneration and cognitive decline [27]. The identification of specific mutations that can cause AD also allowed for the development of cellular and animal models to study the pathophysiological alterations that link these mutations to neurodegeneration.

Despite a less aggressive progression, SAD shares the major characteristics of FAD, including Aβ plaque deposition and NFT pathology, and accounts for the vast majority (>95%) of all AD cases [28]. While no SAD-causative mutations have been found, a considerable number of genetic loci that increase or decrease the risk for developing SAD have now been reported [29–34]. The first identified was the *APOE* locus, encoding apolipoprotein E (APOE) [35]. The *APOE2*, *APOE3* and *APOE4* alleles correspond to APOE with cysteine at amino acid positions 112 and 158, cysteine at 112 and arginine and 158, or arginine at 112 and 158, respectively. Compared to the major *APOE3* allele, *APOE2* has been reported to be protective, while *APOE4* increases late-onset AD risk by ~three-fold for heterozygous carriers and 15-fold for homozygous carriers [36]. Despite its partial penetrance, the relatively high frequency of *APOE4* in the general population (~13%) makes it the single largest cause of AD [37]. APOE is most studied as a lipid carrier secreted from astrocytes that facilitates Aβ clearance from the brain, however, recent studies have revealed potentially detrimental roles of APOE4 also in neurons and microglial cells [38, 39]. Genome-wide association studies (GWAS) in the last decade have identified numerous additional SAD risk genes, many of which are expressed primarily in non-neuronal cells of the brain [29–34] (Table 1). Combined with the persistent failure of AD clinical trials, largely aimed at reducing Aβ production by neurons, these recent genetic findings have begun to shift the focus of AD research toward better understanding the roles and functions of non-neuronal cells during neurodegeneration in AD. In addition, while FAD appears to be caused primarily by overproduction of Aβ, it has become clear that other mechanisms, including defective clearance or aberrant degradation of Aβ, are likely to be important drivers of many SAD cases.

**Table 1:** Alzheimer’s disease risk genes

				Human Brain FPKM				
Gene	Mutation type	Molecular function	% identity	Neurons	Astrocytes	Microglia	Oligodendrocytes	Endothelial
**APP**	**Coding**	**Integral membrane protein**	**97.3**	**160.9**	**16.6**	**6.9**	**109.5**	**41.0**
**PSEN1**	**Coding**	**Protease**, γ**-secretase complex**	**92.7**	**5**	**7.4**	**5.7**	**16.5**	**2.7**
**PSEN2**	**Coding**	**Protease**, γ**-secretase complex**	**96**	**1.1**	**1**	**0.3**	**0.3**	**1.3**
ABCA7	Both	ATP-binding cassette transporter	76.4	0.1	0.1	0.1	0.1	0.1
ACE	Non-coding	Metalloprotease	82.8	0.1	0.1	0.1	0.1	0.4
ADAM10	Non-coding	Metalloprotease	95.9	9.1	7.4	22.6	15.6	6.3
ADAMTS1	Non-coding	Metalloprotease	80.8	3.5	0.9	0.1	9.7	3.1
APOE	Coding	Lipoprotein	71.7	0.5	3.3	0.5	0.2	0.1
BIN1	Non-coding	Endocytic adaptor	95.6	1.2	0.9	6.9	6.7	1.7
CASS4	Non-coding	Tyrosine kinase docking	63.1	0.3	0.2	8.5	0.3	1.5
CD2AP	Non-coding	Scaffolding, actin cytoskeleton	86.5	1.4	1.7	7.9	1.4	4
CD33	Non-coding	Surface receptor	39.0^a^	0.1	0.2	9.5	1	0.1
CELF1	Non-coding	RNA-binding protein	99.6	7.9	6.3	9.5	4.9	3.1
CLU	Non-coding	Extracellular chaperone	76.6	19.3	384.2	0.5	9.6	15.6
CR1	Non-coding	Surface receptor	48.9	0.1	0.1	0.2	0.1	0.1
EPHA1	Non-coding	Receptor tyrosine kinase	87.2	0.1	0.1	0.1	0.1	0.1
FERMT2	Non-coding	Extracellular matrix scaffolding	98.2	3.3	43.2	2.9	6.9	6.8
HLA-DRB1	Non-coding	Antigen presentation	58.9^a^	0.6	1.1	27.1	3	0.7
INPP5D	Non-coding	Phosphatidylinositol phosphatase	87.5	0.1	0.5	18.9	1.4	2.3
IQCK	Non-coding	Calmodulin-binding domain	71.8	4.7	20.8	0.2	6.4	0.6
MEF2C	Non-coding	Transcription factor	93.5	51.9	3.8	44	3.9	4.1
MS4A6A	Non-coding	Transmembrane protein	53.7^a^	0.1	0.7	24	5.2	0.1
PICALM	Non-coding	Endocytosis, clathrin assembly	96.5	12.3	14.6	65.6	37.3	20.5
PTK2B	Non-coding	Tyrosine kinase	95.3	2	0.9	1.3	0.4	0.8
SLC24A4	Non-coding	Na^+^/K^+^/Ca^2+^ exchanger	94.4	1.9	0.2	0.5	0.2	0.1
SORL1	Non-coding	Endocytic receptor/sorting	93.2	9.9	17.9	78.9	6.7	0.8
SPI1	Non-coding	Transcription factor	87.9	0.1	0.1	0.3	0.1	0.1
TREM2	Coding	Surface receptor	50.6	0.1	0.4	27.1	1.4	0.4
TXNDC3	Non-coding	Thioredoxin domain	63.5	0.1	0.1	0.2	0.2	0.1
WWOX	Non-coding	Oxidoreductase	93.7	4.6	5.1	1.1	1.8	0.8
ZCWPW1	Non-coding	Zinc finger domain	60	0.2	0.5	0.5	0.7	0.1

FAD causative genes are shown in bold, SAD risk genes in normal font [20–22, 29–32, 34, 35]. Percent amino acid identity of mouse to human orthologue is indicated (ensembl.org [46])

^a^Indicates multiple orthologues. Fragments Per Kilobase of transcript per Million mapped reads (FPKM) of AD-linked genes from purified human brain cell types are also indicated (brainrnaseq.org [226])

## Alzheimer’s disease models

As the most common neurodegenerative disorder, AD has been studied intensively. Valuable contributions to our understanding of this disease have been made in numerous different systems, including post-mortem patient brain samples, human and animal cell lines, as well as invertebrate, zebrafish and rodent models of disease. Post-mortem brain samples are vital for identifying the cellular and molecular changes associated with neurodegeneration, but provide no ability to alter or intervene in the course of disease. Cultured human and rodent cells have been useful for examining the effects of Aβ and other disease-related molecules on cellular function and health, however as dividing cells they do a poor job of modeling neurodegeneration and many other age-related aspects of AD. Further, while primary brain cells can be cultured from rodents, studies using human cells have utilized mostly non-neuronal cell lines. Non-mammalian models overexpressing disease-related proteins can also serve as in vivo screening systems to identify conserved mechanisms of toxicity or protection, however, the considerable evolutionary distance between these models and humans limits their utility.

Mice have formed the backbone of AD research for a quarter century due to the powerful genetic toolkit available and comparatively close evolutionary relationship with humans [40]. Mice possess most of the same major brain regions and neurotransmitter systems as humans, and are uniquely amenable to rapid assessment of neuronal and circuit function, as well as cognitive performance.

Mouse models that successfully mimic age-dependent neurodegeneration rely mostly on neuronal overexpression of human proteins carrying FAD-causing mutations [41, 42]. For instance, 5xFAD mice use the mouse *Thy1* promoter to overexpress human APP harboring three disease-associated mutations (the Florida (I716V), London (V717I), and Swedish (K670N/M671L) mutations) together with PSEN1 harboring two FAD-linked mutations (the L286V and M146L mutations) [43]. However, this need to express mutated human proteins, typically at considerably higher levels than their endogenous counterparts, in order to achieve a neurodegenerative phenotype hints at some of the limitations of mice as an AD model. In fact, across neurodegenerative diseases, mouse mutations corresponding to human disease-linked mutations rarely result in neurodegenerative phenotypes. This may be due in part to the fact that age is the single greatest risk factor for neurodegeneration and mice have much shorter lifespans than humans. There may also be intrinsic differences in the resilience of mouse and human neurons in the face of oxidative stress, pathologic protein aggregates or other perturbations, though the mechanisms are not fully understood.

In addition to the well-established FAD models, mouse models of numerous SAD genes have also been developed. At many disease-associated loci, the exact mutation(s) linked to AD risk have not been identified, necessitating examination of SAD risk gene knockout mutations [31]. As these mutations alone generally do not cause neurodegeneration, examining their effects on pathology has typically been done in the context of established FAD-mutation mouse models. This approach has identified numerous mechanisms by which SAD risk genes impact AD pathology and has broadened our appreciation of the contributions of non-neuronal cell types to brain health and neurodegeneration. Despite this progress, our understanding of the effects of SAD risk mutations, and of the exact roles that each cell type plays during neurodegeneration, remains far from complete.

In the face of the immense impact that mouse models have made to our understanding of AD mechanisms and pathophysiology, it is also important to acknowledge the potential caveats of mouse studies. While mice are much closer evolutionarily to humans than most other genetic model systems, there remain considerable differences in the functions of proteins, signaling pathways, cellular processes and the interactions between different cell types when comparing the two species [44, 45]. At the protein level, this is highlighted by examining the amino acid sequence identity of different proteins that either cause, or alter the risk for, AD (Table 1). The largely neuronally expressed FAD proteins are nearly identical between human and mouse, exhibiting greater than 90% amino acid identity. In stark contrast, the proteins encoded by a number of SAD risk genes, including the microglial cell surface proteins TREM2, CD33, CR1 and MS4A6A, are only about 50% identical between human and mouse, comparable to the difference between human and insect presenilin proteins [46]. These cross-species differences may also extend more broadly to cell types, such that mice are generally a better model for understanding neuronal phenomena than they are for processes occurring in innate immune cells such as microglia.

While the exact reasons remain uncertain, the sobering reality is that hundreds of clinical trials over more than 30 years have failed to provide an effective treatment that alters the course of neurodegeneration in AD [47]. Hope remains that earlier detection of at-risk individuals, enabling earlier intervention with existing therapeutics, will eventually yield benefit in AD [9]. However, the field has already begun to embrace new ideas—in particular a more balanced view of brain function and dysfunction, acknowledging the importance of multiple brain cell types and their complex, interconnected functions [8]. Another avenue of potentially critical importance is the emerging ability to model human disease using human cells that is becoming increasingly feasible with developing iPSC technologies.

## Induced pluripotent stem cells

**Fig. 2:**
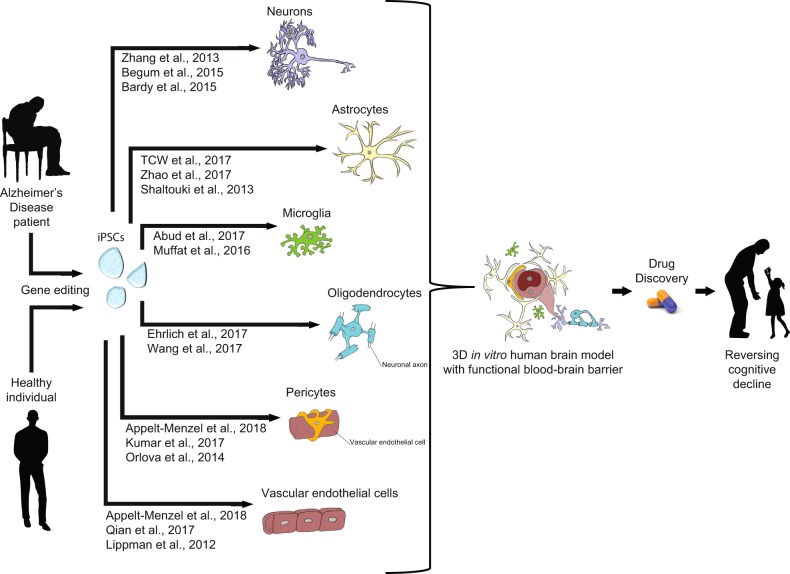
Human iPSC differentiation to brain cell types. Somatic cells from patients or healthy individuals can be reprogrammed to iPSCs and subsequently differentiated into all major brain cell types for in vitro studies. Such studies can examine cellular functions as well as how they are impacted by AD hallmark pathologies or AD-linked mutations. Genome editing techniques can be used to introduce or correct AD-linked mutations to examine phenotypes in isogenic backgrounds. 3D and co-culture models allow for examination of interactions occurring between cell types and sub-types to better model processes occurring in vivo. These and developing techniques hold promise for better understanding the relevant pathomechanisms underlying AD, and will hopefully facilitate development of effective therapeutics to combat dementia

Since the first publication describing how to generate iPSCs from human somatic cells in 2007, there has followed an explosion of methods to direct differentiation into the varied cell types of the body, including brain cells [48–52]. Methods of inducing neural progenitor cells (NPCs), numerous neuron subtypes, as well as astrocytes, microglia, oligodendrocytes, endothelial cells, and pericytes have all been established (Fig. 2). Further, co-culture models incorporating multiple brain cell types have been developed as first steps towards better modeling the complex interactions that occur between these cells in vivo. In all cases, the research community is continuing to refine these methods. Differentiation protocols continue to increase the yield, purity, and maturity of brain cell types. Three dimensional (3D) and co-culture models are still developing, but even now can be utilized to generate AD hallmark pathologies that do not arise in 2D monocultures. Further improving these models to facilitate more meaningful neurodegenerative disease research will be a major goal.

As age is the primary risk factor for AD and other neurodegenerative disorders, it may seem counterintuitive to study AD using stem cells. It is worth noting though that even in the early stages following differentiation, neurons derived from AD patient iPSCs, and from iPSCs carrying FAD mutations, generally exhibit AD-related phenotypes such as elevated Aβ production [53–55]. These early alterations presumably parallel the understudied early stages of disease progression that occur in vivo. 3D cultures derived from the same sources allow for the age-dependent accumulation of Aβ and tau aggregates on a timescale of months [56, 57]. While still far short of the timeline for development of pathology in the human brain, such cultures can be maintained for years in vitro if desired, in line with the lifespans of laboratory mouse strains [58, 59]. Importantly, unlike most other AD model systems, such cultures do not require exogenous overexpression of mutant proteins for the development of disease-relevant pathologies. Thus, while iPSC models are far from perfect, they provide a number of advantages over other systems that are likely to facilitate novel insights into AD pathomechanisms.

Initially, iPSC lines derived from healthy individuals and from patients exhibiting a given disease were isolated, differentiated into the cell type(s) of interest, and compared. A plethora of iPSC lines have been generated from early and late-onset AD patients—as well as healthy age- and sex-matched individual—and numerous repositories have been established around the world to make such lines available to researchers. Comparing cells derived from healthy vs. diseased individuals can identify cellular phenotypes associated with a disease state that may transcend individual genetic differences. Indeed, as GWAS have shown, many different genetic alterations can contribute to the development of AD, so in some cases shared cellular phenotypes can unify disparate genetic changes [8]. Generating iPSC-derived brain cells from a specific individual also has potential applications for personalized medicine and could eventually allow for identification and treatment of patient-specific alterations underlying disease [60, 61].

Genetic diversity, however, can also be a major hinderance to experimental analysis. When trying to understand the function of a specific gene or mutation for instance, additional genetic differences may mask or exacerbate a phenotype. Thus, tools for manipulating gene expression and generating mutations in iPSCs and their derivatives have been critical developments in the field, allowing for introduction or correction of specific mutations without altering the remaining genetic background. The CRISPR/Cas9 system has been revolutionary in this regard, allowing for targeted mutagenesis and base pair resolution editing of eukaryotic genomes [62–64]. While care needs to be taken to rule out off target mutations generated by CRIPSR/Cas9 mutagenesis, this technique allows for examination of the effects of targeted mutations in an otherwise identical (isogenic) genetic background. Genome editing can be used to introduce disease-associated mutations into iPSC lines from healthy individuals, or to correct mutations in cell lines from diseased patients. The nuclease-dead Cas9 (dCas9) system further allows for targeted repression or activation of gene expression in a temporally controlled and reversible manner without the need of gene editing [65, 66]. Complementing examinations of patient-derived iPSC lines, these techniques have begun to yield novel findings about the pathological mechanisms underlying AD, and promise to push forward research in the field of neurodegeneration and beyond.

## Human neural progenitor cell and neuron models

As non-dividing cells, neurons face considerable challenges in maintaining their health and proper function over a lifetime that can span many decades. Indeed, the numerous neurodegenerative diseases that afflict humanity attest to these challenges and speak to the importance of better understanding both the changes that occur during aging, as well as the mechanisms that normally help to ensure neuron health and survival. iPSCs can be differentiated into NPCs, which can subsequently be patterned to different neuronal lineages [67–69]. Both passive and directed differentiation protocols having been developed for numerous different neuron subtypes, including glutamatergic, GABAergic, cholinergic and dopaminergic neurons, though existing protocols are biased strongly towards excitatory neurons [53, 68–75]. Early studies served primarily to validate the iPSC-derived models themselves and to test whether these models recapitulated findings from the large body of AD literature based on human post-mortem brain samples, rodent and other studies (Table 2 highlights select studies of AD using iPSC-derived cells). Consistently, human neuronal function and survival are compromised by treatment with exogenous Aβ [76–82]. Similarly, NPCs and neurons derived from SAD and FAD patients exhibit elevated Aβ_42_ production and/or Aβ_42_/Aβ_40_ ratio as well as increased tau phosphorylation [38, 53–55, 83–93]. Studies using iPSC-derived neurons also suggest neuron sub-type differences in Aβ secretion and susceptibility to Aβ-induced toxicity that vary between glutamatergic and GABAergic neurons, as well as between glutamatergic neurons expressing markers of different brain regions [79, 83, 94].

**Table 2:** Select important AD studies utilizing iPSC-derived brain cells

Model	Reference	Mutation(s)	Significance
Neurons	Israel et al. [89]	sAD, APP^Dp^	Early study of iPSC modeling; elevated Aβ, p-tau, endosome accumulation in AD neurons
	Shi et al. [98]	Down syndrome	Elevated Aβ secretion, Aβ aggregation, tau phosphorylation in DS neurons
	Wang et al. [39]	Isogenic APOE3, APOE4 and APOE null	Elevated Aβ and p-tau levels, GABAergic neuron degeneration in APOE4 neurons; identification of small molecule APOE4 structure corrector
Astrocytes	Oksanen et al. [128]	Isogenic PSEN1^ΔE9^	Increased Aβ production and oxidative stress, altered cytokine release and Ca^2+^ homeostasis, reduced neuronal support function in PSEN1 astrocytes
	Lin et al. [38]	Isogenic APOE3 and APOE4	Impaired Aβ clearance and increased cholesterol content of APOE4 astrocytes
Microglia	Lin et al. [38]	Isogenic APOE3 and APOE4	Reduced Aβ uptake from media and fAD organoids, reduced morphological complexity of APOE4 microglia
3D cultures	Choi et al. [56]	Overexpression of APP^K670N/M671L^, APP^V717I^, PSEN1^ΔE9^	Robust deposition of Aβ and filamentous tau in vitro; demonstrates that Aβ can cause tau deposition
	Park et al. [170]	Overexpression of APP^K670N/M671L^, APP^V717I^, PSEN1^ΔE9^	Triculture model system incorporating iPSC-derived neurons, astrocytes, and immortalized human microglia; recapitulates AD phenotypes, microglial recruitment, and neuroinflammation

Numerous studies have focused on characterizing additional effects of FAD-linked mutations on induced neurons. Glutamatergic neurons expressing mutated APP^V717I^ were used to demonstrate that this amino acid substitution alters APP subcellular distribution and cleavage by both β- and γ-secretases, underlying the perturbed Aβ production and elevated tau phosphorylation observed in these cells [92]. In contrast, β-secretase-mediated cleavage of APP, and Aβ production, were reduced by the protective APP^A673T^ mutation [95]. Neurons carrying APP duplications exhibited more and larger early endosomes, while those harboring the APP^V717I^ or APP^K670N/M671L^ mutations exhibited reduced mitophagy and defects in low density lipoprotein endocytosis, indicative of functional impairment in cellular uptake, trafficking and degradation pathways [89, 90, 96]. APP^E693Δ^ neurons further exhibit endoplasmic reticulum and oxidative stress [78]. iPSC lines from Down’s syndrome (DS) patients have also been studied as a model of AD. DS patients develop early onset AD, thought to result from triplication of the *APP* gene as part of trisomy 21 [97]. DS iPSC-derived neurons show elevations in Aβ secretion and phosphorylated tau similar to that caused by FAD-linked mutations [98–101]. Intriguingly, deletion of the supernumerary copy of APP from DS cells was able to restore Aβ production to control levels and correct many of the gene expression alterations caused by trisomy 21, but was not able to restore altered tau phosphorylation, indicating Aβ-dependent and independent phenotypes in DS [101].

AD-linked mutations in *PSEN1* and *PSEN2* have also been modeled in iPSC-derived glutamatergic neurons, revealing a wide range of phenotypes. Isogenic PSEN1^ΔE9^ and PSEN1^null^ iPSC-derived neurons were used to test whether this AD-linked PSEN1 mutation acts via loss- or gain-of-function, demonstrating in fact, a gain of γ-secretase function without loss of other functions [90]. iPSC-derived neurons carrying PSEN1^A246E^, PSEN1^V89L^, and PSEN1^L150P^ mutations, like neurons derived from SAD-patient cells, were also more sensitive to Aβ-induced toxicity and oxidative stress than cells from healthy individuals [54, 102]. Furthermore, multiple defects in cellular trafficking and degradation pathways have been documented in AD patients and animal models [103–105]. Consistently, fibroblasts and iPSC-derived neurons from multiple PSEN1-mutation carriers show defects in autophagic, ubiquitin proteasome and endo-lysosomal degradation pathways, as well as in mitophagy [55, 87, 106, 107]. Potentially arising from defective mitophagy, mitochondrial dysfunction, elevated oxidative stress and oxidative damage have also been described in PSEN1^P117L^- and PSEN1^A246E^-carrying human cells [107, 108]. Elevated apoptotic cell death and DNA damage response pathway activation was additionally observed in neurons carrying AD-linked PSEN1 mutations [55, 84]. Furthermore, NPCs carrying PSEN1^S169del^ and PSEN1^A246E^ mutations exhibited accelerated differentiation into glutamatergic neurons, though it remains unknown whether this affects the functional properties of mature neurons [84]. Interestingly, iPSC-derived cholinergic neurons carrying the PSEN2^N141I^ mutation exhibited reduced excitability and impaired insulin-induced Ca^2+^ influx compared to isogenic controls, suggesting a functional defect consistent with that found in AD patients [53, 85, 109].

Patient-derived SAD cells often exhibit many of the same phenotypes as cells bearing FAD mutations when differentiated into neurons. In addition to elevated Aβ levels and tau phosphorylation, iPSC-derived neurons from SAD patients can exhibit enlarged endosomes, mitochondrial dysfunction, activation of ER, and oxidative stress pathways, elevated DNA damage as well as increased sensitivity to Aβ toxicity and oxidative stress [54, 78, 89, 110, 111]. Altered gene expression, early neuronal differentiation and maturation, as well as perturbed activity of the transcription factor REST have also been observed [112]. Importantly, these phenotypes are not observed in cells derived from all SAD patients, reinforcing the genetic heterogeneity of SAD [78, 89, 102, 110, 111, 113].

In some cases, the effects of SAD risk genes have also been modeled. The first identified SAD risk factor, *APOE4* has been the most studied risk mutation in iPSC-derived cells, as it has been in rodent and other models. iPSC-derived neurons carrying APOE4 produce more Aβ and have higher levels of tau phosphorylation compared to APOE3 cells [35, 38, 39, 110, 114]. Surprisingly, elevated p-tau levels were not dependent on Aβ in APOE4 cells, indicating perturbation of additional pathways regulating tau phosphorylation [39]. APOE4 also induced endosome abnormalities, defects in autophagy and mitophagy, and widespread gene expression alterations in neurons [38, 96, 106, 112]. Interestingly, synaptic structure and function were also altered by the presence of APOE4, with glutamatergic neurons exhibiting more synaptic sites and increased frequency of spontaneous miniature synaptic transmission compared to isogenic APOE3 controls [38]. APOE4 GABAergic interneurons experienced degeneration in culture (though glutamatergic and dopaminergic neurons did not), while cholinergic neurons exhibited elevated sensitivity and altered Ca^2+^ signaling in response to glutamate toxicity [39, 110]. Intriguingly, iPSC-derived excitatory neurons lacking the SAD risk factor CLU were less sensitive to Aβ-induced toxicity, consistent with *CLU* single nucleotide polymorphisms being associated with reduced AD risk [31, 115]. Sullivan and colleagues took a broader approach, performing a shRNA knockdown screen of more than 50 AD candidate genes in iPSC-derived neurons, examining effects on Aβ secretion and tau phosphorylation [116]. Identifying the SAD risk factor FERMT2 as a modifier of both cellular phenotypes, the authors targeted FERMT2 with CRISPR/Cas9 mutagenesis, confirming that reducing its levels in neurons can decrease Aβ secretion and tau phosphorylation [116]. Future studies using iPSC-derived neurons will examine phenotypes associated with additional AD-risk genes, and promise to identify mechanisms underlying their roles in AD risk.

## Astrocytes

The most abundant cell type in the human brain, astrocytes, provide physical, energetic, metabolic, and trophic support to neurons and other brain cells [117, 118]. They play important roles in vasomodulation, inflammation and wound healing. Astrocytes also interact closely with neurons and their synapses; astrocytic calcium signalling is regulated by various neurotransmitters and calcium waves can propagate over considerable distances via gap junctions. Astrocytes can regulate neuronal excitability and synaptic transmission via modulation of ion concentrations and by regulating the uptake and recycling of neurotransmitters such as glutamate and GABA [119, 120]. In a neurodegenerative milieu, astrocytes can proliferate and enter a reactive state that is potentially toxic to neurons [121]. Further, astrocytes are a major source of cholesterol and other lipids that are critical for many cellular functions, as well as lipoproteins such as APOE, which are thought to be important regulators of brain Aβ clearance and degradation [122].

Multiple different protocols for differentiation of iPSCs into astrocytes have been developed [123–127]. Astrocytes generated from patients carrying both FAD-linked PSEN1^M146L^ and SAD-linked APOE4 mutations exhibited reduced morphological complexity and altered localization of marker proteins, indicating similar effects of FAD and SAD mutations [126]. PSEN1^ΔE9^ astrocytes showed elevated release and reduced uptake of Aβ_42_, altered Ca^2+^ homeostasis, increased reactive oxygen species production, altered cytokine release and impaired fatty acid oxidation [128, 129]. iPSC-derived astrocytes have also been shown to promote the survival, maturation and function of co-cultured human neurons, effects that can be impaired by PSEN1^ΔE9^ and APOE4 mutations [128, 130, 131]. Further, compared to isogenic APOE3 astrocytes, APOE4 cells showed extensive gene expression alterations, cholesterol accumulation, and reduced ability to internalize Aβ_42_ [38]. APOE4 astrocytes were also found to express and secrete lower levels of APOE protein, which additionally exhibited reduced lipidation, confirming earlier findings from mouse studies [38, 131]. While additional FAD and SAD-linked mutations remain to be examined, these findings indicate considerable overlap in the effects of PSEN1 and APOE mutations on human astrocyte function.

## Microglia

Microglia are the resident innate immune cells of the brain. They play numerous support roles in the developing, adult and aging brain, ranging from synaptic pruning, to clearance of dying cells and other debris, to the regulation of neuroinflammation [132]. Their involvement in neurodegenerative processes has long been appreciated, but until recently microglia were generally thought to respond to disease-associated pathologies rather than to play a causative role [133]. Improved sequencing technologies in the past 10 years have allowed GWAS to identify a surprising number of microglial genes as risk factors for late onset AD, including *TREM2*, *CD33*, *HLA-DRB1*, *INPP5D*, *MS4A6A*, *CASS4,* and *SPI1* [29–32]. These findings clearly implicate microglial dysfunction as a driver of neurodegeneration under certain circumstances.

Protocols for the differentiation of iPSCs into microglia have only recently become available, and will undoubtedly be utilized more heavily to examine the effects of AD-linked mutations in the coming years [134–140]. Induced microglia derived from healthy patients are capable of synaptic pruning, phagocytosis and Aβ uptake, they secrete diverse cytokines and exhibit altered gene expression in response to treatment with exogenous Aβ [134, 135]. Microglia induced from SAD-patient iPSCs were found to have altered phagocytosis and elevated release of certain cytokines following treatment with lipopolysaccharide [139]. Lin et al. compared APOE4 microglia to isogenic APOE3 controls, finding extensive gene expression alterations suggestive of a pro-inflammatory phenotype, reduced morphological complexity, as well as an impaired ability to internalize Aβ from culture media and to clear Aβ aggregates from 3D cerebral organoids [38]. Recent studies have also examined microglia derived from patients carrying non-AD-linked TREM2^W50C^ and TREM2^T66M^ mutations, finding reduced viability under stress conditions and impaired phagocytosis of certain substrates [138, 141]. AD-linked TREM2 mutations, as well as those of other FAD and SAD-linked genes, await examination in iPSC-derived microglia. The findings of such studies could be of particular interest given the significant differences in sequence similarity, and potentially protein function, between the human and mouse orthologues of many microglial disease genes.

## Oligodendrocytes

The primary role of oligodendrocytes is generation of the myelin sheath that wraps the axons of many nerve cells, together forming the white matter of the central nervous system [12, 142]. The myelin sheath, composed primarily of lipids, electrically insulates axons to prevent ion leak and promote rapid propagation of electrical signals over distance. This insulator property contributes to fast excitatory and inhibitory synaptic transmission that underlies cognitive function and coordinated movement. Additionally, oligodendrocytes provide trophic support to neurons, can mediate inflammation and contribute to the regulation of metabolic waste in the brain [12, 142]. AD patients and mouse models exhibit loss of white matter and oligodendrocyte dysfunction early in disease progression, likely contributing to neuronal dysfunction and degeneration [143, 144]. Consistently, multiple AD risk genes play important functions in oligodendrocyte biology. Protocols for differentiation and induction of oligodendrocytes from iPSCs exist, and induction of oligodendrocytes and myelination in 3D organoid models has also recently been reported [136, 145–150]. However, these human systems have not yet been utilized to examine the effects of AD-related pathologies on myelination and oligodendrocyte function.

## The blood–brain barrier

The human brain is a highly vascularized and energetically demanding organ, containing more than 600 km of blood vessels that supply oxygen, glucose, and nutrients to support brain functions, while facilitating removal of carbon dioxide and other metabolic wastes [151]. At the same time, the cerebrospinal fluid (CSF) that bathes the brain has a distinct chemical composition essential for proper neuronal function, and molecules, cells and pathogens carried by the blood can cause brain inflammation and toxicity [19]. Thus, systemic blood is separated from the brain and CSF by the BBB, a specialized structure formed of VECs, pericytes and astrocyte end-feet; this barrier restricts transit of many molecules between the two compartments [19]. BBB dysfunction and breakdown are observed in AD and multiple other neurodegenerative diseases, exacerbating not only brain inflammatory responses, but also impairing blood flow, the delivery of oxygen, glucose and nutrients, as well as the clearance of Aβ and other neurotoxic substances from the brain [152]. Further, the BBB can act as an impediment to the transit of many therapeutics into the brain, so the development of models to test the passage of potential treatments preclinically is of interest [152].

While BBB dysfunction is involved in AD pathophysiology, the exact mechanisms by which it occurs, as well as the potential roles of AD-linked mutations, remain largely unknown. In vitro BBB models have been established using rodent and human primary cells, and in recent years have begun to incorporate human iPSC-derived cells [153, 154]. iPSC-derived brain endothelial cells have been used either alone, in combination with iPSC-derived neural cells and astrocytes, or together with rodent primary pericytes and astrocytes for such models [155–159]. However, while pericyte differentiation protocols exist, no BBB model incorporating all of iPSC-derived endothelial cells, pericytes and astrocytes has yet been reported [160, 161]. The development of a functional BBB model will be of interest not only for screening potential therapeutics to better predict their entry into to the brain, but also to understand whether mutations linked to neurodegenerative disease predispose to BBB dysfunction.

## Co-culture, 3D culture, and in vivo systems

It has become increasingly clear in recent years that multiple different brain cell types can contribute to AD progression [8]. Thus, examining their interactions and impacts on each other is of critical importance; indeed, the ability to do this may be the single greatest strength of AD animal models, and the greatest weakness of in vitro systems. As such, considerable efforts have been made to build models incorporating multiple iPSC-derived brain cell types. In addition, 3D neuronal models, either alone or together with other brain cell types, have allowed for a more faithful recapitulation of Aβ plaques and NFTs, AD hallmark pathologies that are rarely observed in 2D cultures [56, 57, 162]. Together, these advances are allowing the field to more and more accurately model AD using human cells in vitro.

3D tissue culture has a long history, but a landmark for studies of the nervous system was the first description of “cerebral organoids” derived from human iPSCs in 2013 [163, 164]. This approach utilizes the self-organizing properties of iPSCs and their progenitors after embedding in Matrigel, a complex mixture of extracellular matrix and other secreted proteins. The resulting organoids can exhibit regionalization and organized expression of layer- and brain region-specific markers [163]. Organoids grown from iPSCs derived from SAD, FAD or DS patients exhibit elevated Aβ production, the formation of Aβ aggregates, increased tau phosphorylation and altered early endosome markers [57, 165]. Similar 3D culture models—made from NPCs overexpressing FAD proteins with AD-linked mutations—demonstrate bonafide Aβ plaques and NFTs, and demonstrate in human cells that Aβ pathology can drive tau phosphorylation and aggregation [56]. Neurospheroids, made either from NPCs overexpressing mutant FAD proteins, or iPSCs derived from SAD patients, can also recapitulate AD phenotypes and exhibit similar proteomic changes to AD patients [166, 167].

Such neuron-based 3D culture models often also develop astrocytes during the course of development, though they exhibit a paucity of other brain cell types [38, 58, 168, 169]. To study both neuron and astrocyte function, organoids derived from isogenic APOE3 and APOE4 iPSCs were compared; age- and APOE4-dependent elevations of Aβ and phosphorylated tau were found to corresponded temporally to the appearance of astrocytes, thus indicating the critical importance of APOE to astrocytic function [38]. The same study further incorporated iPSC-derived APOE3 and APOE4 microglia into organoids derived from FAD patient cells to monitor microglial effects on amyloid pathology, finding that after 30 days of co-culture organoids with APOE3 microglia exhibited fewer Aβ puncta than those containing APOE4 microglia [38].

A human triculture model incorporating NPC-derived neurons and astrocytes, together with immortalized human microglia, has also been developed to study how interactions between these three cell types affect hallmark AD pathologies and neuroinflammation [170]. Similarly, a recent study reported an organoid model that exhibits spontaneous development of microglial cells [171]. Additional recent studies demonstrate methods to induce oligodendrocytes and myelination in organoid systems [148, 149]. Another intriguing development involves the fusion of organoids initially directed toward excitatory or inhibitory neuronal fates to model the developmental migration of inhibitory neurons and functional integration of these two key neuronal sub-types [172–174]. 3D co-culture models such as these provide an opportunity to examine the effects of AD-linked mutations present in one cell type or sub-type on phenotypes of other brain cells, as well as potential interactions that may occur between different AD-linked mutations. Ultimately such models could incorporate multiple different neuronal sub-types, together with oligodendrocytes, astrocytes and microglia, as well BBB components and vasculature to create a simplified “brain in a dish”.

An additional strategy to examine iPSC-derived brain cell phenotypes and cell-cell interactions is their incorporation into the nervous systems of living rodents. In order to avoid immunogenic rejection of the graft, host strains must lack an adaptive immune system, as in the case of the SCID and Rag2^−/−^ mouse strains [175, 176]. Xenografting of iPSCs—transplanting human iPSCs into mice—has been performed for iPSC-derived neurons and microglia [135, 177, 178], as well as for whole 3D organoids that exhibited functional integration with in vivo neural circuits and vasculature [179]. Such iPSC “xenocultures” open a new window to study in vivo cell type specific interactions using human cells during aging and neurodegeneration.

## Challenges and future directions

The development of iPSC technologies provides the attractive possibility of using differentiated human cells as platforms for drug and mutagenesis screening, reviewed by Elitt and colleagues [180]. Multiple small-scale compound screens using differentiated neuronal subtypes have already targeted disease-related pathways in AD [39, 181–184], Parkinson’s Disease [185], Huntington’s Disease [186], and frontotemporal dementia [187]. A recent effort screened over 1600 compounds for reductions of tau phosphorylation in FAD neurons, identifying numerous hits and ultimately moving forward our understanding of the biology underlying p-tau accumulation [188]. shRNA screening has also been used to test the effects of knocking down more than 50 different AD candidate genes on Aβ and p-tau levels in iPSC-derived neurons [116]. Thus far such screens have targeted primarily neuronal cells, however, human glial cells will also be utilized as screening platforms going forward. Refining high-yield 3D culture techniques to facilitate screening of multiple co-cultured brain cell types holds strong potential to become the gold-standard for future central nervous system drug development. Proof-of-principle has already been demonstrated in a landmark paper that recapitulated the pathology of Zika virus infection in 3D organoids, including a small-scale screen to alleviate its symptoms [189].

Human stem cell technologies also raise intriguing possibilities for regenerative and personalized medicines, topics that have been reviewed elsewhere [60, 61, 190, 191]. A serious safety concern for regenerative medicine, however, is genomic instability exhibited by iPSCs, which can also be an issue for experimental studies using these cells [192–197]. Uncertainty remains about whether iPSCs are actually intrinsically more unstable than other cultured cells, but regardless, care should be taken to limit passage numbers and regularly check iPSC lines used for research for the presence of genomic alterations [198]. While genomic alterations in iPSCs have been reduced by replacing retroviral expression of somatic cell-to-iPSC reprogramming factors with integration-free delivery systems, additional avenues to reduce genomic instability remain a topic of active research [192, 193, 199].

Continuing to improve iPSC differentiation protocols, expanding the repertoire of brain cell sub-types that can be generated, as well as developing more complex 3D co-culture systems to model brain development and disease remain major goals of iPSC researchers. These efforts strive to improve the ease and speed of differentiation protocols, but importantly also the quality, purity and maturity of differentiated cells. Most iPSC differentiation protocols generate heterogeneous populations of the target cell type and various precursors, either necessitating analysis of impure cultures or use of additional methods to purify cell samples such as fluorescence activated cell sorting. This can be particularly problematic for neuron sub-types as even partially mature neurons are extremely sensitive to dissociation and sorting methods. Transcription factor-induced differentiation protocols hold promise to improve both the speed and conversion rate of iPSC-derived cells and have emerged for a number of brain cell types [71, 74, 75, 145, 200].

As a new and still developing field, improving the consistency and reproducibility of iPSC-derived cell types also remain major challenges. A wide range of protocols for differentiation to brain cell types are in use by different laboratories around the world, and even within a given laboratory. Indeed, even using the same differentiation protocol, considerable variability in cellular morphology and gene expression signature can occur between sites [201], with clear potential to affect experimental findings. Furthermore, variability between different clones derived from the same parental iPSC line have also been reported [202]. As such, better standardization of differentiation techniques and growth conditions, more thorough reporting of methodologies and adoption of stringent statistical analyses should help to minimize such variability. To this end, minimal standards for quality control have been suggested, in particular for large repositories of iPSC lines, in order to reduce experimental variability [203, 204]. Such measures include regular testing for donor identity and genetic integrity, testing for microbial contaminations and a standardized nomenclature for genetic modification of iPSC lines.

The ability to generate mature iPSC-derived brain cells that properly mimic their in vivo counterparts is another significant challenge. The various cell types of the brain grow and mature together, often relying on signals from other cell types to help shape their identity. Indeed, significant differences in the transcriptomes of human and mouse glial cells arise following purification from the brain and culture in vitro [205–208]. Consistently, iPSC-derived brain cells and 3D cultures often exhibit similarity to immature cells from the human nervous system [58, 209]. A better understanding of the identity and timing of key signals will enable researchers to better recapitulate the brain milieu in monocultures in vitro. Further, co-cultures incorporating multiple brain cell types, as well as utilization of extended culture times, will help to facilitate the maturation of each cell type so they more closely resemble their counterparts in the adult human brain [58].

3D co-culture systems will be increasingly important iPSC-derived models for neurodegeneration research. In addition to aiding the maturation of brain cell types, these systems promote development of AD hallmark pathologies that are generally not found in 2D cultures [56]. They further provide a platform to better understand the complex interactions and interrelated functions of brain cell types that have been both difficult to model in vitro and difficult to disentangle in vivo. The ability to introduce a specific brain cell type, carrying a mutation of interest, into an otherwise functioning co-culture system provides a powerful tool to isolate cell type- and mutation-specific effects that could be masked otherwise. Indeed, even an analysis of cell-type specific transcriptomic alterations in the presence of an AD-linked mutation in one cell type could be highly informative. Importantly, such a system would also allow for examination of potentially synergistic interactions between different AD risk mutations, an area that remains very poorly studied, but that is likely an important factor in the development of SAD in human patients.

Despite their obvious utility, iPSC-derived 3D co-culture systems remain very much works in progress. An ideal system would include multiple neuronal sub-types, each type of glial cell as well as BBB components, however, in practice reduced systems modelling only some aspects of brain function are likely to be more tractable in many cases. Regardless, continued improvement of brain cell-type differentiation protocols, definition of optimal media and conditions for co-culture experiments as well as identification of better substrates and scaffolding matrices for 3D brain cultures all hold promise for improving these models. The introduction of vasculature, either by self-organizing properties of BBB cells, or by other means, is also of great interest. Organoids often exhibit some degree of cell death and dysfunction in their deeper layers; a functional vasculature to facilitate nutrient, oxygen and waste exchange would likely improve the health of cells in 3D culture. The use of miniature spinning “bioreactors” to provide constant movement of culture media can also circumvent some of these issues and improve the homogeneity of organoid cultures [210, 211].

The ability of iPSC-based models to mimic diseases of aging such as AD has also been questioned, in part due to the loss of age- and environment-dependent cellular and epigenetic signatures that occurs during iPSC reprogramming [212–215]. Such epigenetic de-differentiation is undesirable when attempting to recapitulate disease-related phenotypes in vitro and also limits the application of iPSCs to personalized medicine. One way to overcome this loss of aging signatures is to bypass the iPSC stage with direct reprogramming, whereby fibroblasts and certain other somatic cells can be differentiated directly into neurons or other cell types to study neurodegenerative disease [216–222]. Directly reprogrammed cells retain many age-related cellular and transcriptomic alterations, and in at least some cases are better able to model age-related disease than iPSC-derived neurons [223]. Thus far methods for direct differentiation of neurons, neural precursor cells, astrocytes and oligodendrocytes have been described [217, 218, 221, 222]. Direct reprogramming also has limitations, in particular a relatively poor reprogramming efficiency and low yield of reprogrammed cells [224], however, continued refinement of direct induction methods should allow access to additional brain cell types and better facilitate the study of brain cells with intact aging signatures.

A final frontier of in vitro brain modeling is the generation of coordinated neuronal activity. While iPSC-derived neurons and 3D models exhibit synaptic activity, and connectivity between inhibitory and excitatory neurons has been reported, they do not possess coordinated circuit functions [172–174]. AD patients and rodent models demonstrate widespread defects in circuit properties involving multiple neuronal subtypes [9]. Indeed, recent work demonstrates the potential for therapeutic benefit that can be derived by modulating higher order brain rhythms, controlled by GABAergic interneurons, in AD mouse models [225]. Examination of such circuit-based mechanisms in human iPSC-derived systems would be of great interest, but will require continued efforts to optimise differentiation protocols to generate neuronal sub-types, improve available tools to modulate and monitor neuronal cells in culture, as well as to devise strategies to integrate different neuronal sub-types together to form functional circuits.

## Conclusions

In little more than a decade since the advent of human iPSC technologies we have developed the ability to generate all of the main brain cell types from pluripotent cells. Increasingly complex 3D co-culture systems are also emerging that allow us to reconstitute many of the key interactions between brain cells. These technologies have already contributed greatly to our understanding of human development and human disease, including neurodegenerative disorders such as AD. As these techniques continue to be refined to better mimic in vivo conditions, our ability to model AD using human cells will improve. The functions of SAD risk genes in iPSC-derived brain cell types remains almost completely unexplored, and even the effects of FAD-linked mutations outside of neurons are mostly uncertain. Further, our understanding of how such mutations, either alone or in in combination, affect the interactions between the different cell types of the brain is in its infancy. iPSC-derived brain cell types hold considerable promise for allowing us to answer these questions, and ultimately to identify and implement effective treatments for AD.
